# RNA-Seq based selection signature analysis for identifying genomic footprints associated with the fat-tail phenotype in sheep

**DOI:** 10.3389/fvets.2024.1415027

**Published:** 2024-09-30

**Authors:** Hossein Abbasabadi, Mohammad Reza Bakhtiarizadeh, Mohammad Hossein Moradi, John C. McEwan

**Affiliations:** ^1^Department of Animal and Poultry Science, College of Aburaihan, University of Tehran, Tehran, Iran; ^2^Department of Animal Sciences, Faculty of Agriculture and Natural Resources, Arak University, Arak, Iran; ^3^Invermay Agricultural Centre, AgResearch, Mosgiel, New Zealand

**Keywords:** RNA-Seq datasets, selection signatures, fat deposition, thin- and fat-tailed sheep, SNP calling

## Abstract

Understanding the genetic background behind fat-tail development in sheep can be useful to develop breeding programs for genetic improvement, while the genetic basis of fat-tail formation is still not well understood. Here, to identify genomic regions influencing fat-tail size in sheep, a comprehensive selection signature identification analysis was performed through comparison of fat- and thin-tailed sheep breeds. Furthermore, to gain the first insights into the potential use of RNA-Seq for selection signature identification analysis, SNP calling was performed using RNA-Seq datasets. In total, 45 RNA-Seq samples from seven cohort studies were analyzed, and the F_ST_ method was used to detect selection signatures. Our findings indicated that RNA-Seq could be of potential utility for selection signature identification analysis. In total, 877 SNPs related to 103 genes were found to be under selection in 92 genomic regions. Functional annotation analysis reinforced the hypothesis that genes involved in fatty acid oxidation May modulate fat accumulation in the tail of sheep and highlighted the potential regulatory role of angiogenesis process in the fat deposition. In agreement with most previous studies, our results re-emphasize that the *BMP2* gene is targeted by selection during sheep evolution. Further gene annotation analysis of the regions targeted by the sheep evolution process revealed that a large number of genes included in these regions are directly associated with fat metabolism, including those previously reported as candidates involved in sheep fat-tail morphology, such as *NID2*, *IKBKG*, *RGMA, IGFBP7, UBR5, VEGFD* and *WLS*. Moreover, a number of genes, including *BDH2*, *ECHS1*, *AUH*, *ERBIN* and *CYP4V2* were of particular interest because they are well-known fat metabolism-associated genes and are considered novel candidates involved in fat-tail size. Consistent with the selection signature identification analysis, principal component analysis clustered the samples into two completely separate groups according to fat- and thin-tailed breeds. Our results provide novel insights into the genomic basis of phenotypic diversity related to the fat-tail of sheep breeds and can be used to determine directions for improving breeding strategies in the future.

## 1 Introduction

Archeological evidence suggests that sheep were first domesticated in the Middle East approximately 9,000–11,000 years ago (1). Since then, domestic sheep have spread all over the world, as more than 1,400 breeds have been established through adapting to a diverse range of environments under which they have been reared and bred for various purposes by humans (2). In general, sheep breeds can be divided into two main groups, fat-tailed (short fat-tail, long fat-tail, and fat rumped-tail) and thin-tailed (long thin-tails and short thin-tails), in terms of tail phenotype (3). Since fat-tailed breeds have been criticized for intensive production systems, in terms of economic value and energy cost, there is a need for sheep breeders to understand the genetic mechanisms controlling tail fat development to create breeding programs aimed at reducing tail size (4). However, the genetic basis of such differences is still not well understood. It has been demonstrated that natural and human selection pressures leave detectable signatures in the genome and can be identified by comparing breeds with extreme phenotypes, such as fat- and thin-tailed sheep breeds (5). Hence, these selection signatures are crucial to understand how domestication and sheep breeding shaped the genome structure of fat- and thin-tailed breeds. This can be explored through the investigation of the genomic regions of interest that potentially harbor selective sweeps caused by selection.

A selective sweep is a process that occurs when a particular genetic variant or allele rapidly becomes more common in a population due to natural or artificial selection (6). The signatures of selection can be detected by examining the distribution of genetic variations surrounding a selected allele, which can lead to the identification of a region with reduced genetic diversity around the selected allele (7). Several attempts have been made to use this approach within the context of fat-tail development (8–16). For instance, two independent experiments were conducted in previous studies utilizing genotype data from Iranian sheep populations and the comprehensive Sheep HapMap project. These experiments compared thin- and fat-tailed sheep breeds, and the analysis confirmed associations with fat deposition in three genomic regions on chromosomes 5, 7, and X across both datasets (10, 11). Also, Ahbara et al. (17) used the Illumina Ovine 50 K SNP BeadChip to identify genetic relationships and candidate regions linked to fat deposition and tail morphology related to 11 Ethiopian sheep populations. They reported several candidate genes such as NPR2, HINT2, SPAG8, and INSR associated with fat deposition, and ALX4, HOXB13, and BMP4 for tail morphology. Genome-wide selection signature analysis revealed eight candidate regions influencing these traits, providing insights into the genetic mechanisms in Ethiopian indigenous sheep.

Genotyping by arrays is challenged by high-throughput sequencing technologies, next generation sequencing (NGS). For instance, SNP arrays only consider a fraction of the genetic variants, a limitation that becomes more pronounced when applied to the analysis of local breeds. The rapid development of NGS has made it possible to identify genetic variants comprehensively and efficiently, which is necessary for detecting genomic selection signatures between different breeds (18). Since most of the previous studies used SNP array to genotype animals, few investigations have been conducted to use whole genome sequencing (WGS) for genomic scanning of selective sweeps between fat- and thin-tailed sheep breeds (19). Conversely, RNA-Seq stands out as one of the most widely employed sequencing applications of NGS and is primarily utilized for analyzing transcriptome data. RNA-Seq captures genome information and can be employed for SNP discovery. The feasibility of uncovering genetic variants from this data was demonstrated in our previous study (4). Therefore, RNA-Seq datasets can be considered a cost-effective alternative to WGS for genetic variant calling (20). In this context, a large number of RNA-Seq datasets have been generated over the last 15 years and provide a yet-unexploited resource of genetic variants in numerous sheep breeds from different populations. Hence, in the present study, we performed a selection signature identification analysis for the first time based on the identified SNPs from seven independent RNA-Seq studies (21–27). The primary goal of this study was to explore the potential of using RNA-Seq datasets for selection signature identification analysis. The secondary goal was to identify genomic regions with strong evidence for selective sweeps through a genomic comparison between fat- and thin-tailed sheep breeds. Assuming that selective sweeps can highlight key genes associated with fat-tail formation, functional enrichment analysis was performed on the genes located in the identified genomic regions. The insights gained in this study May provide a foundation for future research on the genetic improvement and breeding of sheep breeds.

## 2 Materials and methods

### 2.1 RNA-Seq dataset

**Figure 1:**
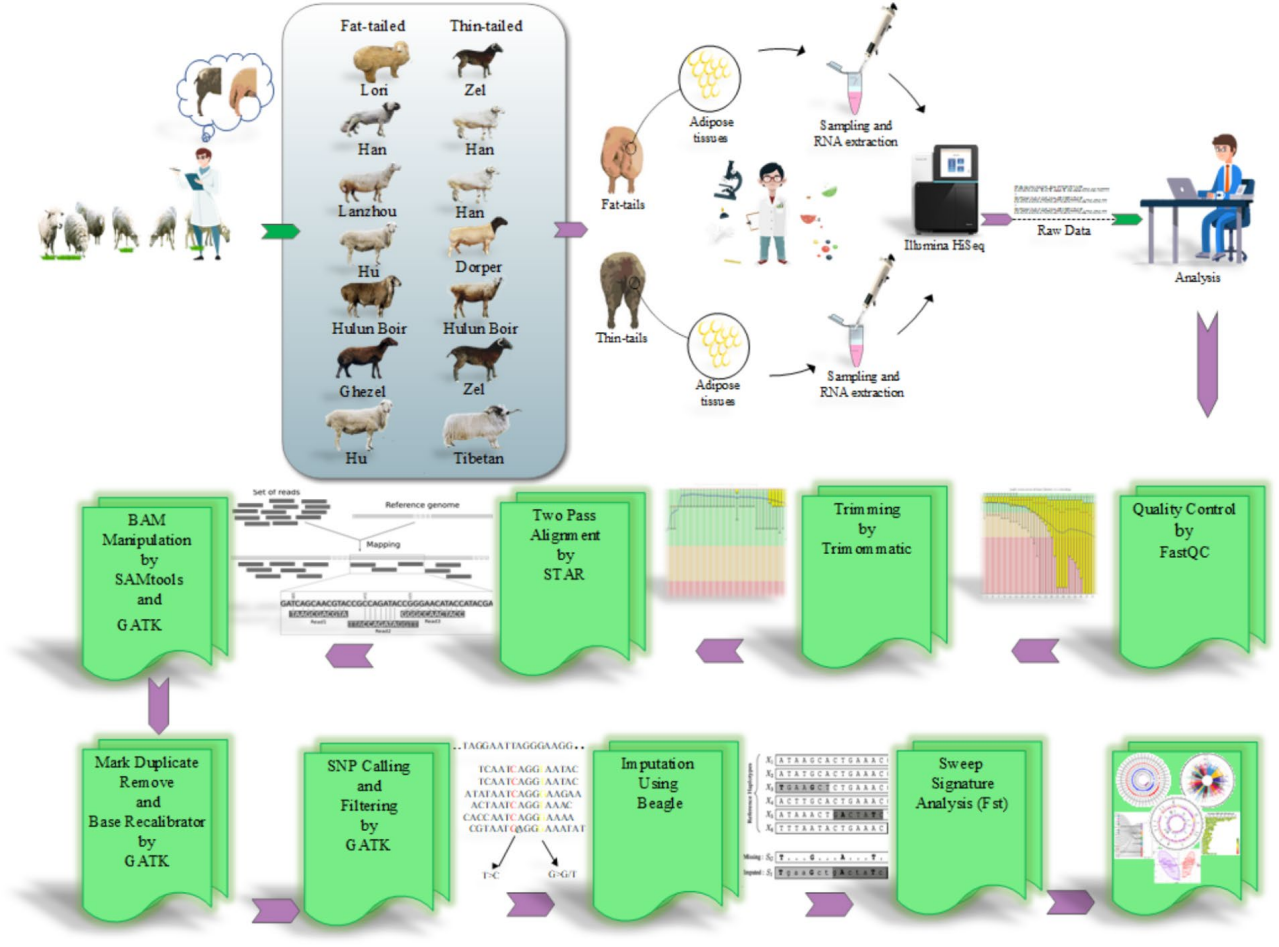
The pipeline used to perform SNP calling from RNA-Seq datasets and selection signature identification analysis.

The RNA-Seq datasets were obtained from studies that focused on extreme phenotype groups of sheep breeds in terms of fat-tail size. Therefore, a comprehensive literature review was performed, and seven studies were found to be consistent with our purpose. A list of these datasets is provided in Table 1. All samples were isolated from the fat-tail tissue of male sheep and were paired-end sequenced, with a minimum of three replications per breed. In total, 45 samples, including 22 fat-tailed and 23 thin-tailed samples, were analyzed from nine breeds. All the data were retrieved from the GEO database of NCBI and were analyzed based on the same bioinformatics pipeline (Figure 1).

**Table 1:** List of datasets used in the present study.

Accession number	Fat-tailed	Thin-tailed	Reference
PRJNA508203	Lori, MEF (4)^a^	Zel, MET (4)	Bakhtiarizadeh et al. (21)
PRJNA699984	Han, CHF (3)	Han, CHT (3)	Guangli et al. (22)
PRJNA432669	Lanzhu, CHF (3)	Han, CHT (3)	Ma et al. (23)
PRJNA745517	Hu, CHF (3)	Dorper, CHT (3)	Yuan et al. (24)
PRJNA517348	Hulun Boir, CHF (3)	Hulun Boir, CHT (3)	Fan et al. (25)
PRJNA598581	Ghezel, MEF (3)	Zel, MET (4)	Farhadi et al. (26)
PRJNA792697	Hu, CHF (3)	Tibetan, CHT (3)	Jin et al. (27)

a Numbers in parentheses indicate biological replicates.

MEF, Middle East Fat-tailed sheep from Middle East; MET, Middle East Thin-tailed sheep from Middle East; CHF, Chinese Fat-tailed sheep; CHT, Chinese Thin-tailed sheep.

### 2.2 Quality control and mapping

To ensure data quality, the RNA-Seq datasets were initially subjected to quality control analysis using the FastQC software (version 0.11.5) (28). Additionally, the Trimmomatic software (version 0.38) was used to remove low-quality reads/bases and adaptor sequences to improve the reliability and accuracy of the data by eliminating undesired artifacts and ensuring that only high-quality reads were retained for downstream analysis (29). The trimming parameters used were as follows: TRAILING: 20, MAXINFO: 120:0.90, and MINLEN: 120 (21). To map the clean reads against the sheep genome (Rambouillet version 1.0.104), STAR software (version a2.7.9) was applied according to a two-pass alignment approach (−-outFilterMismatchNoverLmax 0.06 and –outFilterMultimapNmax 10) (21). In this method, during the alignment process, a new index file is generated that encompasses high-confidence novel splice junctions along with known splice junction sites. Thus, this strategy facilitates alignment to the reference genome considering both known and novel splice junctions (30). The known splice site information was obtained from ENSEMBL GTF file (Rambouillet v1.0.104, release 106) (31). To minimize false positive results, only uniquely and concordantly paired-end aligned reads were retained for downstream analysis. Subsequently, those reads that were mapped to the same location were marked using the MarkDuplicates function of GATK tool (version 4.2.6.1) (32). These reads were considered PCR duplicates and were excluded from further analysis (33). To increase the accuracy of SNP calling, a base quality score recalibration was performed using BaseRecalibrator and ApplyBQSR modules of the GATK tool (34) based on Ensembl ovine SNP database (*Ovis aries*, Rambouillet).

### 2.3 SNP calling and filtering

Finally, the HaplotypeCaller module of GATK was employed to call putative variants for each sample with a stand_call_conf and stand_emit_conf value of 30 and mbq of 25 (4). The identified initial variants were filtered using a universal set of filtering parameters, including HomopolymerRun >5, Total depth of coverage <10, QualitybyDepth <2, RMSMappingQuality <40, MappingQualityRankSum <−12.5 and ReadPosRankSum <−8 (4). To enhance the reliability of the identified SNPs, a further filtering step was implemented, and the variants with at least three supporting reads for the SNP were retained. Furthermore, variants located in problematic regions, including low-complexity regions (simple sequence repeats regions, ± 3 bases) or regions with bidirectional transcription, exonic, and splice signal regions (within 5 bp of the intronic flanking region), were removed (4). Only variants meeting these criteria and reported as known SNPs in Ensembl ovine SNP database were retained for further analysis. This filtering process aimed to improve the quality and reliability of the identified SNPs for downstream analysis.

### 2.4 Imputation

To improve the completeness of genotypic information, genotype imputation was implemented. The reference SNP file related to the ovine reference genome was obtained from Animal-ImputeDB database. The reference panel contained 29,889,815 SNPs from 450 samples related to 43 domestic sheep breeds, ensuring a diverse genetic background as well as providing a high-resolution dataset (35). Since the genomic coordinates of the SNPs in this database were based on a different sheep genome (Oar_v3.1), LiftOver tool (version 46e) (36) was used to convert genomic coordinates from Oar_v3.1 to Rambouillet v1.0.104. To do this, all SNPs were converted to Rambouillet v1.0.104 coordinates with the relevant chain file obtained from the UCSC Genome Browser. Subsequently, the Beagle software (version 5.4) (37, 38) was used based on the haplotype frequency model to impute the SNPs. This model assumes no missing genotype in the reference genotypes to simplify calculations. Genotype imputation relies on identity by descent (IBD), where two parts of the chromosome are inherited from a common ancestor without recombination or crossing over. By accurately identifying IBD segments, untyped alleles in the target haplotype can be copied from the reference haplotype, effectively increase the number of markers (39, 40). SNPs with a prediction probability above 90% (according to DR2 value, squared correlation between imputed and real genotypes) and a MAF > 0.05 (frequency of rare or less common alleles) were retained to optimize imputation accuracy for subsequent analyses (41). To assess the accuracy of the imputed genotypes, a concordance analysis was used. To do this, 1% of the common SNPs between the identified SNPs and the reference file (from Animal-ImputeDB) were randomly selected and masked before imputation. The remaining SNPs were imputed and applied to check whether the masked SNPs were predicted correctly or not (42). This process was repeated five times to ensure the reliability of the results.

### 2.5 Selection signature identification analysis

To detect signatures of selection between the two distinctive groups, two complementary methods, the Fixation index (F_ST_, proposed by Wright) (43) and the unbiased Fixation index (Theta, proposed by Weir and Cockerham) (44), were estimated. To do this, the fat- and thin-tailed breeds were pooled together, and then the F_ST_ and Theta statistics were calculated for these two groups (fat-tailed group: 22 samples and thin-tailed group: 23 samples). These population differentiation indices quantify the level of genetic differentiation between fat- and thin-tailed sheep breeds based on differences in allele frequencies. The values of the fixation indices can theoretically range from 0 (showing no differentiation) to 1 (indicating complete differentiation, i.e., populations are fixed for different alleles). Regions with extreme F_ST_ and Theta values can be considered as good candidates for selective sweeps. In the present study, all extreme windows in the 99.9th percentiles (top 0.1% of F_ST_ and Theta) were selected as harboring the genomic regions with selection signatures. A window of 40 kb was chosen because it appeared to provide a better signal than other arbitrary window sizes (45). Moreover, the genetic relationship between fat- and thin-tailed sheep breeds was explored using principal component analysis (PCA) based on the SNPs located in the genomic regions identified to be under positive selection in thin- and fat-tailed breeds. This analysis aimed to determine how the animals allocated to the groups using these low-density positively selected markers. All scripts for estimating the Wright (F_ST_) and Weir and Cockerham (Theta) indices, as well as PCA, were written and performed in R v 4.0.2.

### 2.6 Functional analysis

The identified candidate regions were annotated using SNPeff tool (version 5.1) (46) to detect the genes that harbored the SNPs in these regions according to a reference genome annotation file (ENSEMBL GTF, Rambouillet v1.0.104, release 106). To gain insight into the potential functions of these genes, functional enrichment analysis was performed using EnrichR tool. This tool is a comprehensive and popular gene set enrichment analysis web-server search engine that enables researchers to analyze and visualize gene lists for enriched terms through connection to other important databases such as GO, KEGG, etc. (47). Biological processes with an FDR <0.05 were considered as significant terms.

## 3 Results

### 3.1 RNA-Seq dataset analysis

**Figure 2:**
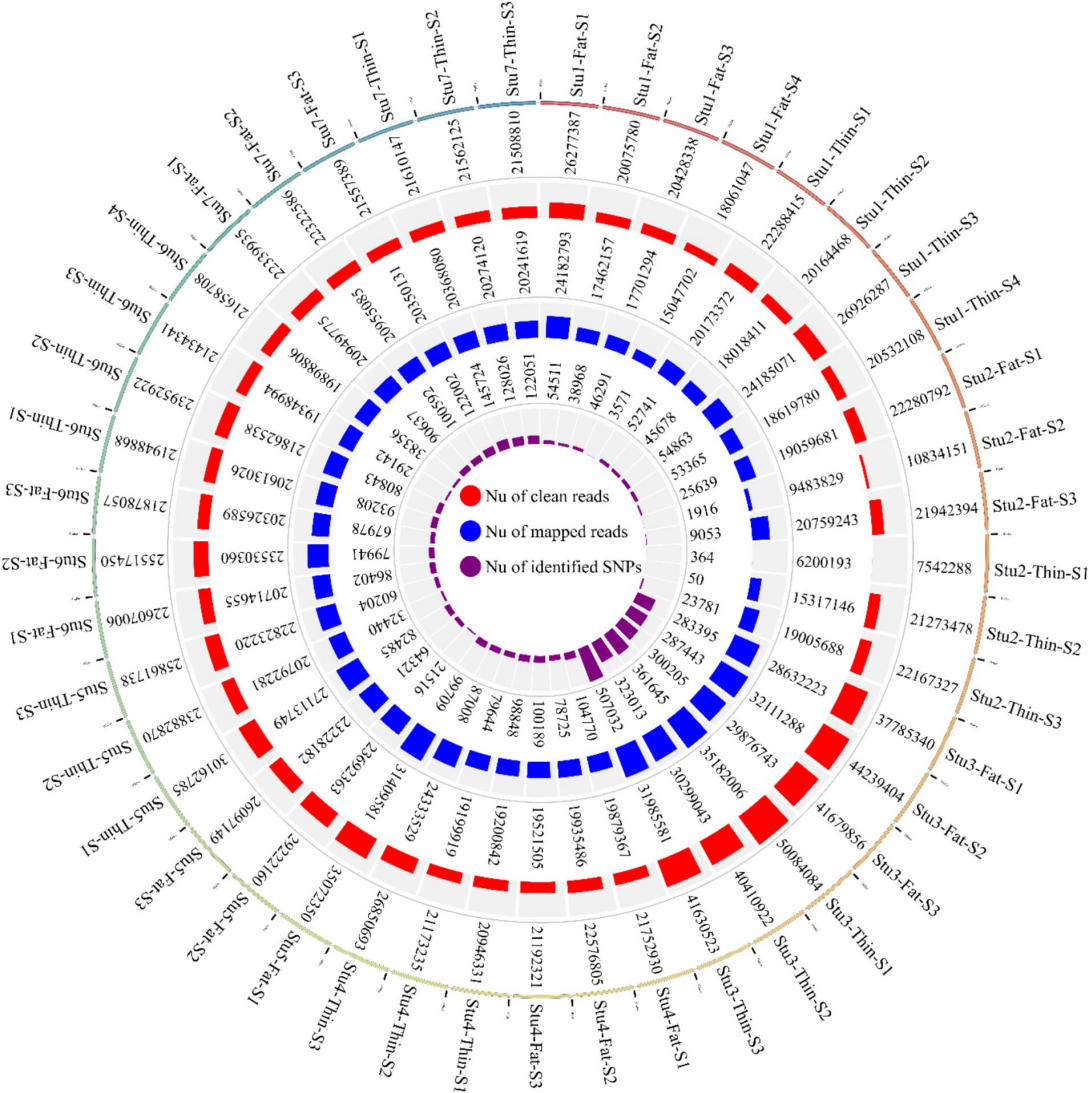
Circos plot indicating the number of clean reads (outer ring), mapped reads (middle ring), and identified SNPs (inner ring) per sample of the seven studies. “Stu” represents the number of studies (Stu1, Study 1), “Fat” and “Thin” represent fat- and thin-tailed breeds, respectively, and “S” represents the number of samples per study. There are 45 samples in total.

In this study, seven RNA-Seq datasets related to 22 fat-tailed and 23 thin-tailed sheep were analyzed. The total number of raw reads obtained for the seven datasets was 1,136,651,699; with an average of 25 million reads per sample. Of these, 556,807,475 and 579,844,224 reads belonged to fat- and thin-tailed breeds, respectively. Following quality control and filtering, 1,131,314,100 reads remained (Figure 2). The clean reads were then aligned to the sheep reference genome, and 973,867,046 reads were successfully aligned to the genome, with an overall alignment rate of 93% (Figure 2). The overall alignment rates between the two groups were similar, with 92.6% ± 0.05 for the fat-tailed breeds and 92.5% ± 0.06 for the thin-tailed breeds. Of the aligned reads, 973,867,046 reads were uniquely mapped to the genome and were retained for further analysis. The average alignment rate of the uniquely mapped reads per sample was 92.6%, ranging from 77.1 to 98.5% (Supplementary material S1).

### 3.2 SNP calling

**Figure 3:**
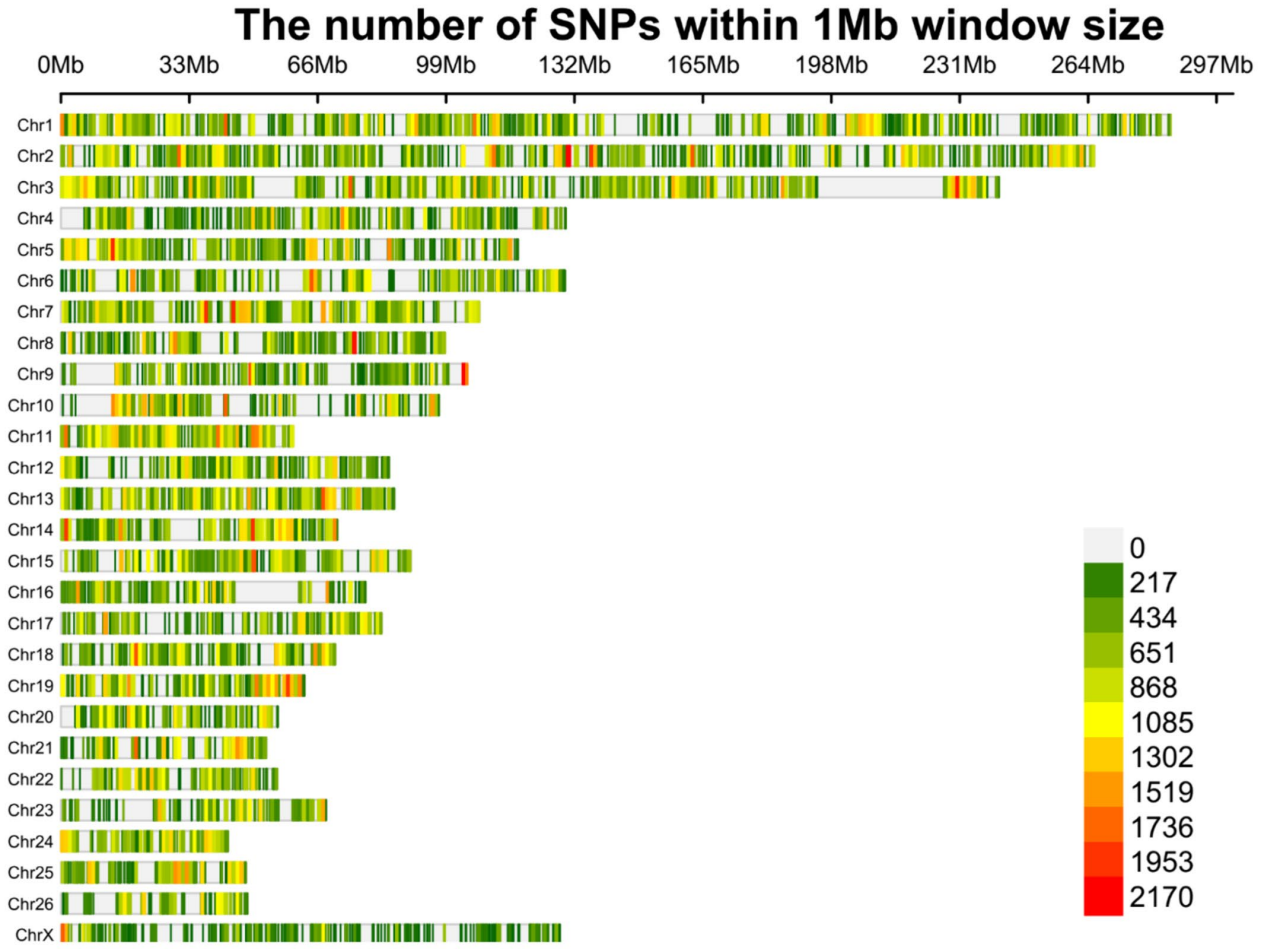
Density plot of the identified SNPs after imputation across chromosomes. Different colors represent the quantity of the SNPs based on the legend.

**Figure 4:**
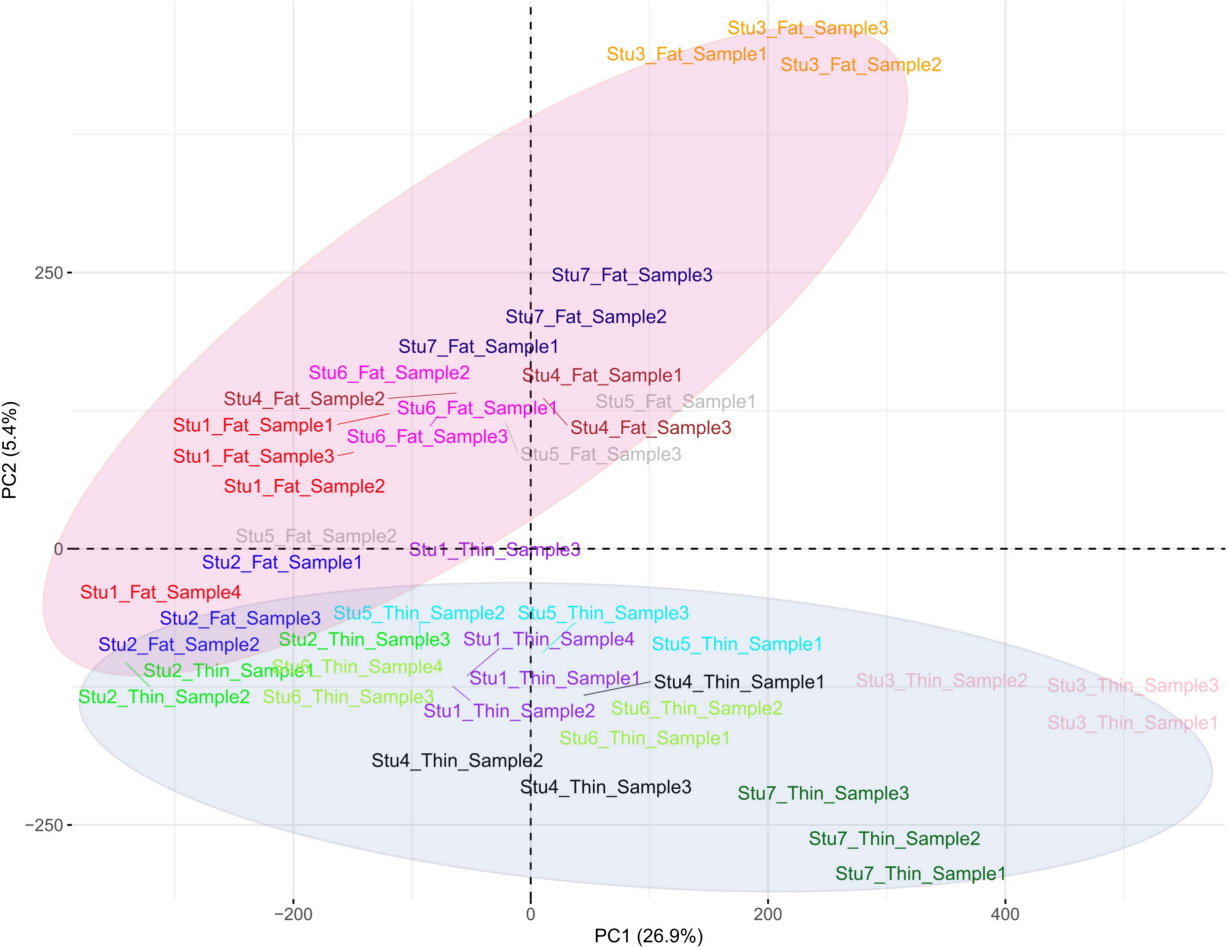
Principal component analysis (PCA) based on all the identified SNPs in thin- and fat-tailed breeds. Individuals are colored based on their breed, and the red and purple colors represent the fat- and thin-tailed sheep breeds, respectively. “Stu” represents the number of studies (Stu1, Study 1), “Fat” and “Thin” represent fat- and thin-tailed breeds, respectively, and “S” represents the number of samples per study.

Overall, 1,719,262 unique biallelic variants were identified across all samples based on our stringent pipeline. Among these, 1,415,762 variants were reported as known SNPs after comparison to the sheep dbSNP database. It is worth emphasizing that only reported SNPs in dbSNP database of ENSEMBL were applied to increase the reliability of the results. The complete list of the identified SNPs per sample is provided in Supplementary material S2. Subsequently, these SNPs were imputed (and filtered with DR2 > 0.90 and MAF > 0.05), and 18,577,286 SNPs were found across samples, including 1,171,918 unique SNPs. Of all SNPs, 8,981,485 SNPs were detected in fat-tailed breeds, while 9,595,801 SNPs were identified in thin-tailed sheep breeds. The average number of detected SNPs per sample in fat- and thin-tailed sheep breeds were 408,249 and 417,208, respectively. The inner ring of the Circos plot in Figure 2 represents the number of identified SNPs per sample of each study. Among the unique SNPs, the highest number of SNPs were found on chromosome 1 (128,394), and the lowest numbers were located on chromosome 26 (16,153). Additionally, 24,977 SNPs were found on chromosome X. Figure 3 displays the density of these SNPs per chromosome. Concordance analysis of the imputation analysis revealed that the imputed genotypes were highly consistent based on all five repetitions, with a concordance rate of 99%. This indicates that the imputed genotypes were recovered with very high accuracy. To investigate the population structure, all the identified SNPs were applied to perform PCA analysis. The first and second principal components (PC1 and PC2) of the PCA differentiated fat- and thin-tailed breeds (Figure 4). In general, the first two PCs explained 32.3% (PC1 = 26.93%, PC2 = 5.4%) of the variation in the entire genomic data.

### 3.3 Selection signature identification analysis

**Figure 5:**
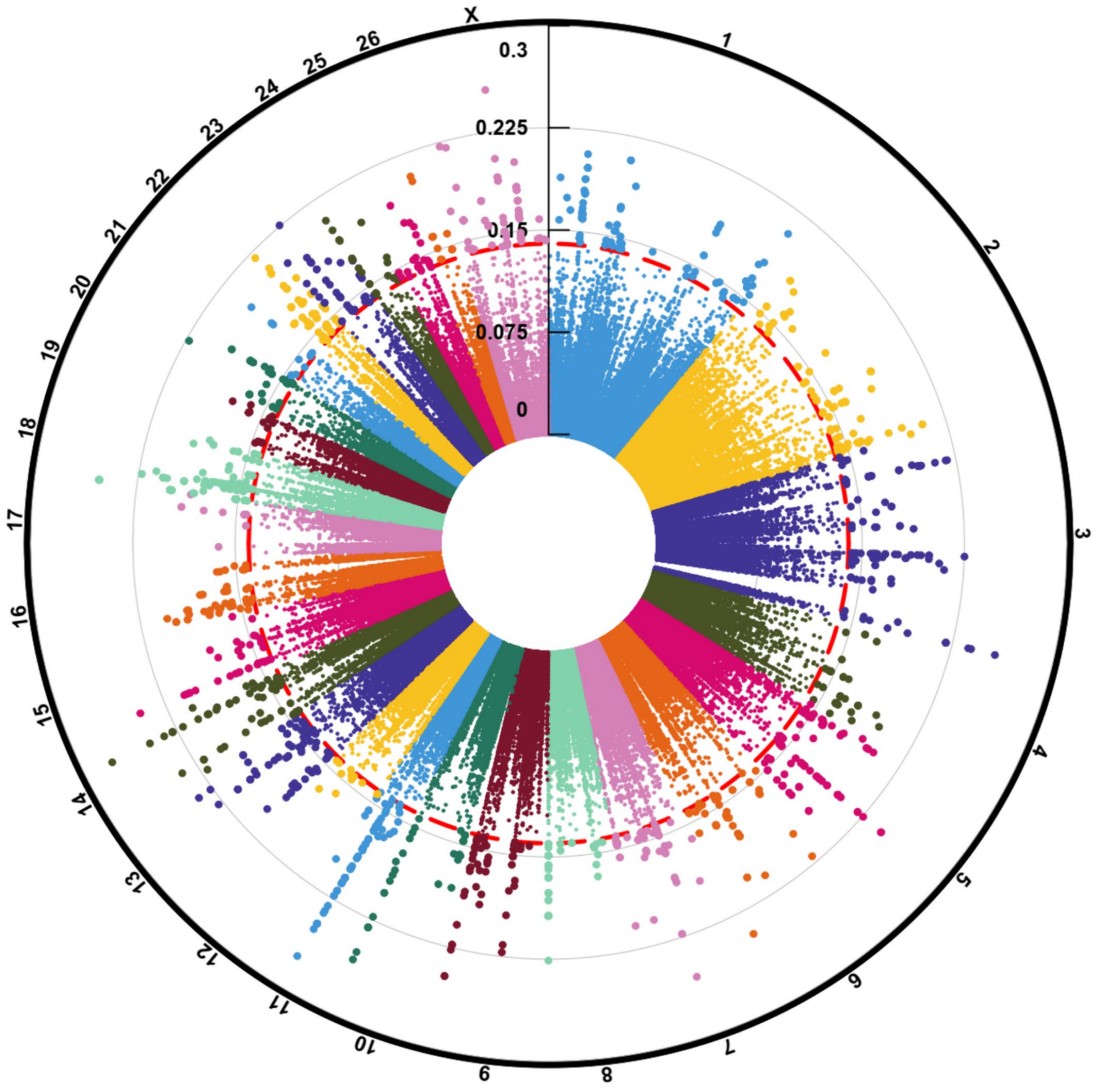
Manhattan plot of the genome-wide selection signature distribution of windowed FST in fat- and thin-tailed sheep breeds. The outermost ring shows chromosome numbers, and the middle red ring marks the 0.1% percentile threshold for F_ST_ > 0.15.

**Figure 6:**
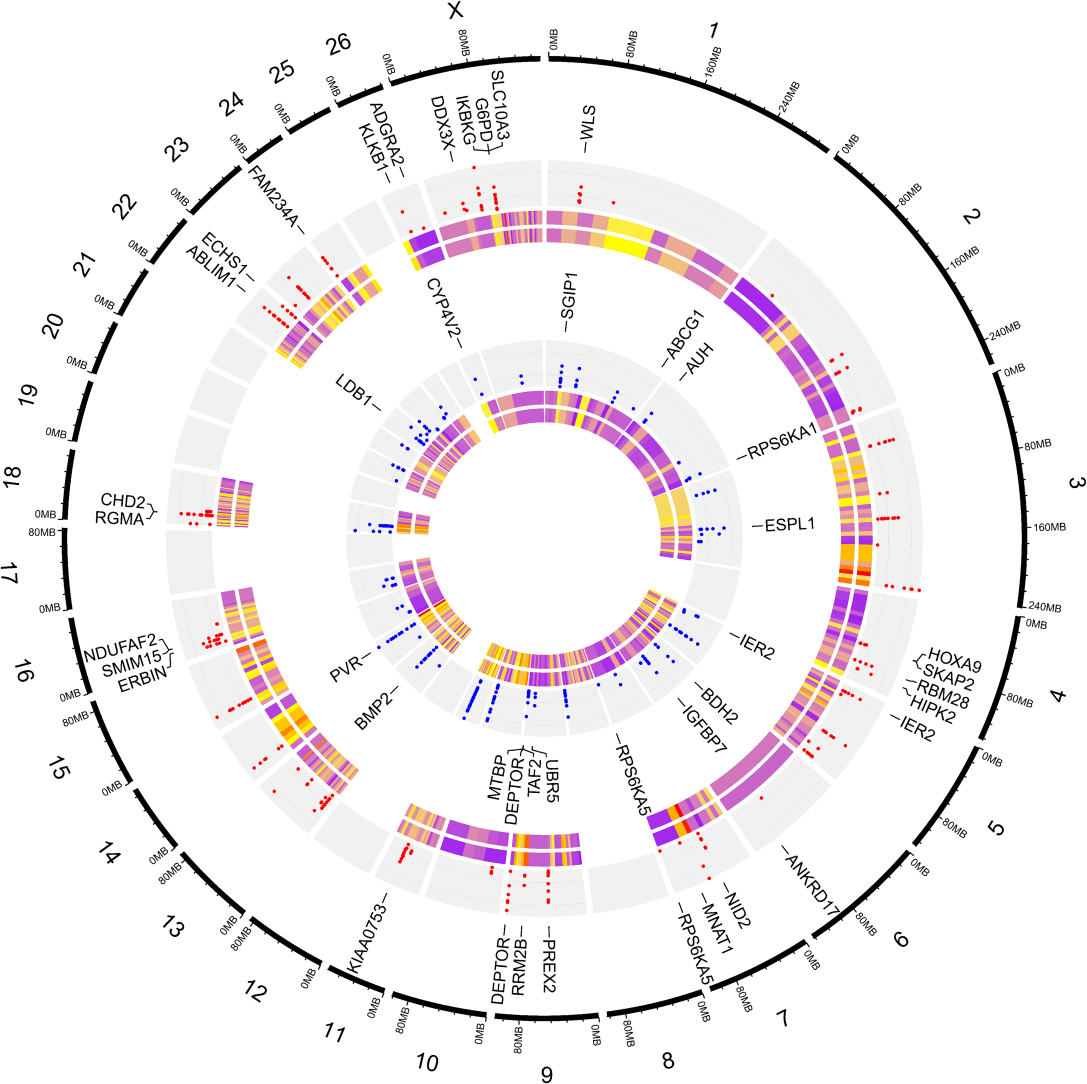
Circos plot related to genome-wide distribution of the putative selection signatures. The outermost ring shows the chromosome numbers. SNPs were divided into two groups based on their heterozygosity levels, as the SNPs that were more heterozygous in thin-tailed sheep breeds are shown in the first (red points) inner gray ring, while the more heterozygous SNPs in fat-tailed sheep breeds are presented in the second (blue points) inner gray ring. The first layer of the heatmap displays the FST values, which ranged from 0.15 (purple) to 0.28 (yellow). The second layer of the heatmap displays the Theta values, which ranged from 0.23 (purple) to 0.42 (yellow). Some of the most important genes associated with fat metabolism are displayed.

**Figure 7:**
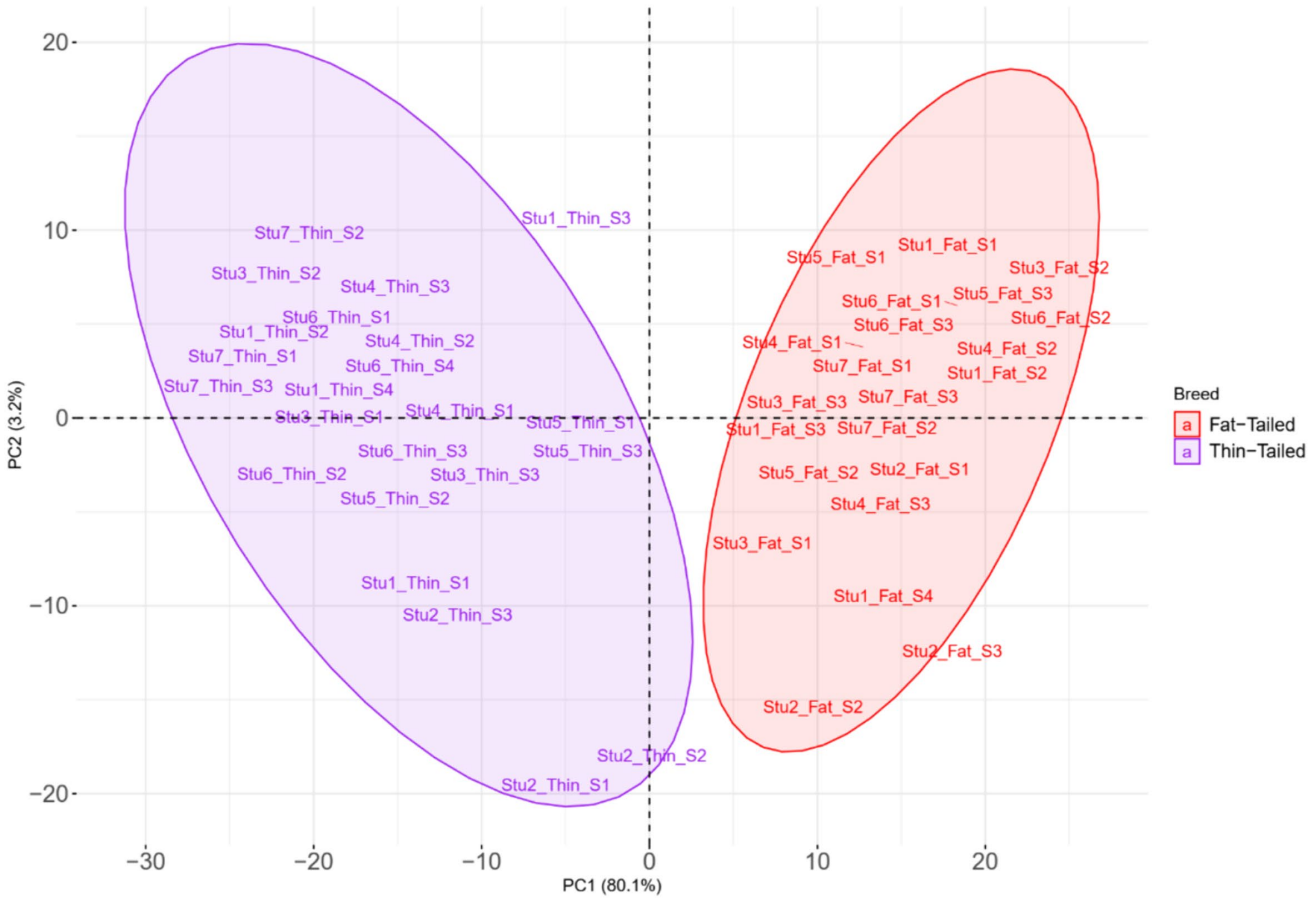
Principal component analysis (PCA) based on the SNPs located in the genomic regions identified to be under positive selection in thin- and fat-tailed breeds. Individuals are colored based on their group (fat- or thin-tailed), and the red and purple colors represent the fat- and thin-tailed sheep breeds, respectively. “Stu” represents the number of studies (Stu1, Study 1), “Fat” and “Thin” represent fat- and thin-tailed breeds, respectively, and “S” represents the number of samples per study.

To elucidate the differences due to selection and identify selected regions and candidate genes involved in fat-tail development, genome-wide pairwise F_ST_ values between fat- and thin-tailed breeds were estimated based on 1,171,918 SNP genotypes (Figure 5). The average distance between adjacent SNPs across the whole genome was 2.35 kb, providing a high resolution for accurately detecting selection signatures. To assess the degree of breed differentiation due to genetic structure, F_ST_ and Theta statistics were estimated, which evaluates the proportion of genetic variance within and between subpopulations. While Wright’s F_ST_ method does not consider sampling error, Weir and Cockerham proposed an unbiased F_ST_ estimator (Theta) that accounts for sample size (11). Pearson’s correlation coefficient between the two methods in our study revealed a strong and significant positive correlation (r2 = 0.97); thus, only the F_ST_ was used for further analysis (Supplementary material S3). The average F_ST_ across the genome was 0.015 (SD = 0.020). Evidence of selection was identified for 877 SNPs within 92 regions, which passed the cutoff (extremely high values in the 0.1% right-tail of the F_ST_ distribution) and were considered regions of the genome that exhibited significant population differentiation. In other words, these regions could be potential candidate regions under positive selection. The F_ST_ values of these SNPs ranged from 0.15 to 0.28, with an average of 0.18. These SNPs were distributed across all chromosomes (except 12, 17, 19 and 25), as the highest and lowest number of SNPs were located on chromosomes 18 (86) and 21 (3), respectively. Additionally, 103 genes, including 87 genes with gene symbol annotations, were identified in these regions; these genes can be regarded as positively selected genes involved in fat-tail development in sheep. The genome-wide distribution of these signatures is shown in Figure 6 as a Circos plot. Additionally, more detailed information on these genomic regions can be found in Supplementary material S4. Using PCA and based on the first two principal components (PC1 vs. PC2), samples were divided into two groups (fat- and thin-tailed), according to the SNPs found in the selection signature regions. The percentages of variance explained by PC1 and PC2 were 80.1 and 3.2%, respectively (Figure 7).

### 3.4 Functional enrichment analysis

**Figure 8:**
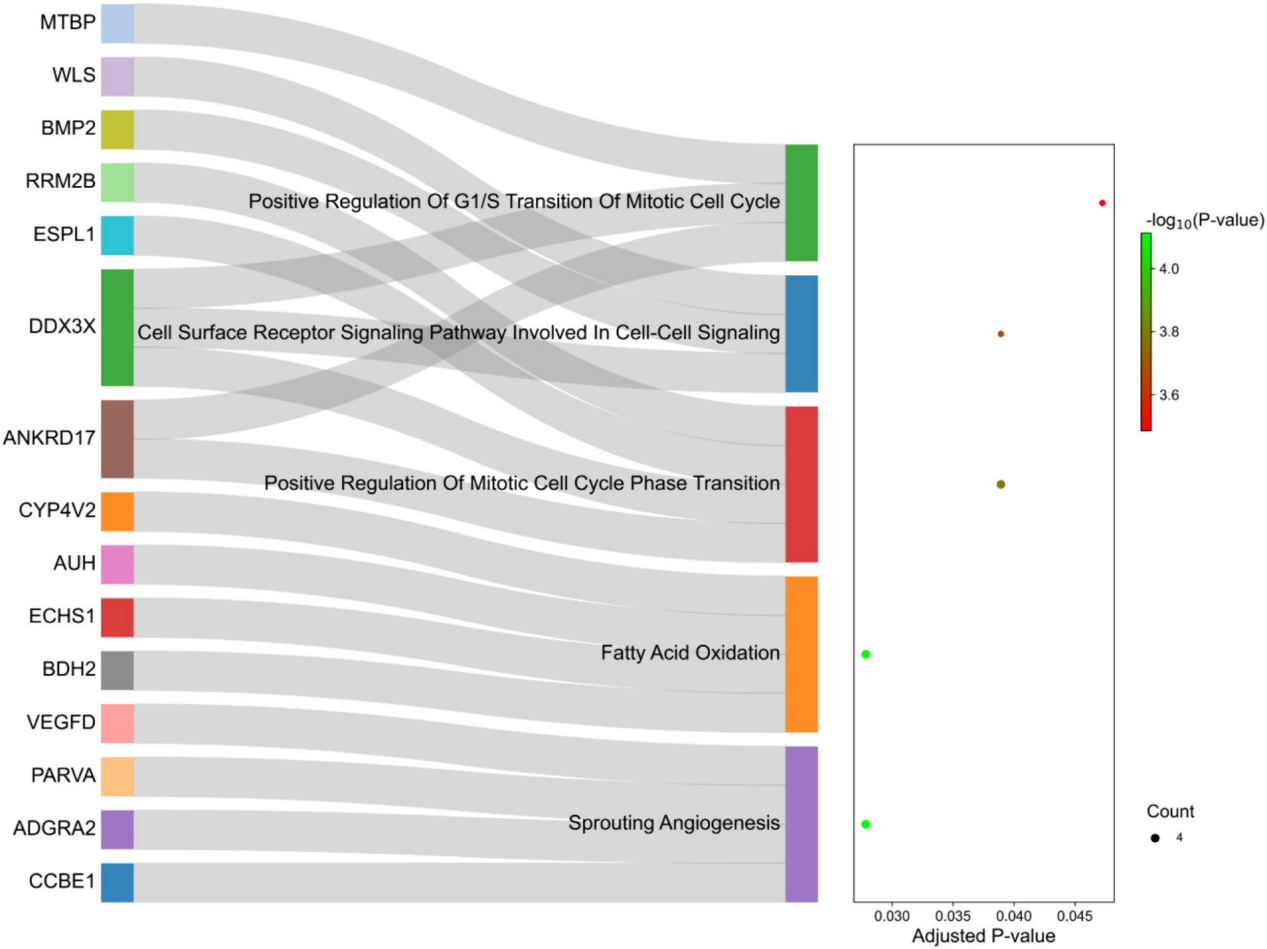
Sankey plot of the functional enrichment analysis related to the genes identified in the selection signature regions. Count represents the number of genes associated with the terms.

The functional interpretation of the genes identified in the selected regions revealed enrichment of 129 biological processes with *p*-values <0.05. As listed in Supplementary material S5, some of the terms were related to fatty acid metabolism, such as “Fatty Acid Beta-Oxidation,” “Fatty Acid Oxidation” and “Fatty Acid Catabolic Process.” Of these, five terms were significant after multiple testing correction by the FDR method including “Sprouting Angiogenesis,” “Fatty Acid Oxidation,” “Positive Regulation Of Mitotic Cell Cycle Phase Transition,” “Cell Surface Receptor Signaling Pathway Involved In Cell–Cell Signaling “and “Positive Regulation Of G1/S Transition Of Mitotic Cell Cycle” (Figure 8). Additionally, 10 genes were directly annotated to be associated with fat metabolism-related processes, including *BMP2, BDH2, ECHS1, ABCG1, AUH, CYP4V2, DDX3X, ERBIN, SLC10A3, WLS* and *IKBKG*, which are labeled in Figure 8.

## 4 Discussion

Domestic animals have been subjected to both natural and artificial selection, which have left distinctive genomic features. One of these features is the reduction of genetic diversity in the regions targeted for specific traits, such as fat-tail shape. In this regard, genome-wide scanning for selection signatures is a powerful method for identifying candidate regions that are subjected to the phenotypic diversity of sheep breeds. Despite numerous studies devoted to identifying signatures of selection in sheep, the genetic basis of various fat-tail shapes in different sheep breeds has not been fully elucidated. To address this gap, discerning genomic variation between fat- and thin-tailed sheep breeds is critical. Evidence of selection signatures offers insights into potential genetic factors related to fat deposition. This difference provides an ideal opportunity to elucidate the molecular mechanisms underlying energy storage and lipid metabolism since sheep can be useful animal models for biomedical research. Taking advantage of the RNA-Seq data, the aim of the current study was to use a comparative genomic analysis between fat- and thin-tailed sheep breeds to identify putative signatures of selection that May be related to fat-tail formation. For this purpose, seven fat-tailed and five thin-tailed sheep breeds were used (Table 1). Notably, these breeds can be used to identify general selection signatures involved in fat deposition in sheep tails based on the hypothesis that organ physiology is conserved across animals. In this context, there are several pathways that are deeply conserved among the species (48, 49). According to this hypothesis, rodents or other animals can be used as models to study human diseases or biological processes. Therefore, genomic signals related to fat deposition are conserved among different sheep breeds and can be inferred by comparing fat- and thin-tailed sheep breeds. The signatures of selection denote the genomic regions influenced by positive selection, resulting in an increased frequency of beneficial alleles within a population.

Various methods have been developed to detect these signatures including allele frequencies based approaches such as F_ST_ (Wright method) and Theta (Weir and Cockerham method) (11) and haplotype-based methods such as haplotype homozygosity score and hapFLK. It is demonstrated that the haplotype-based procedures have greatest power to detect ongoing selection, as they explore the structure of haplotypes and essentially identify unusually long haplotypes carrying the ancestral and derived alleles (50). However, phenotype of sheep tail is a result of completed selection, not an ongoing selection (51). On the other hand, haplotype-based methods need high density SNPs and phasing for haplotype analysis (50), which are not guaranteed by RNA-Seq data. Hence, in the current study, two allele frequencies methods were applied and showed high concordance. The F_ST_ method is particularly suitable for detecting regions that have been under selection for a long time and that have accumulated in different populations. This method captures historical patterns of variation and divergence that have occurred over many generations (52). In this regard, the F_ST_ method was applied in the present study, taking into account from the perspective of population genetics that fat-tailed breeds evolved from their thin-tailed ancestor approximately 5,000 years ago (51). It is worth noting that the number of identified SNPs in thin-tailed breeds (2,498,980) was greater than that in fat-tailed breeds (2,066,424), further supporting this hypothesis (Supplementary material S2).

Over 82% of the variants identified in our primary SNP calling analysis were found in the dbSNP (Ensembl ovine SNP database), emphasizing the high quality and reliability of our SNP genotype data. However, ~18% of the identified SNPs were not available in dbSNP database, which shows that more attempts need to be made to profile the genetic diversity of sheep. Compared to previous studies utilizing whole-genome sequencing data, we identified a significantly lower number of SNPs. This finding is completely in line with this point that most SNPs detected by whole-genome sequencing data are located in intergenic or intronic regions (53–55). Here, to eliminate the variants resulting from RNA editing sites and maximize the reliability of the results, only known SNPs were considered for further analysis (56, 57). In this study, a relatively large number of selection signals were detected (92 genomic regions, Supplementary material S4), reflecting the complexity of the fat-tail phenotype. However, some of these signals May be related to other traits, and further investigations are needed. The highest number of selection signatures was found on chromosomes 18, X and 11 (Supplementary material S4), which suggests greater selection pressure on these chromosomes.

Functional annotation of the identified selection signature regions was performed to investigate their specific role in fat deposition in the tail of sheep. The two most significant terms were “Fatty Acid Oxidation” and “Sprouting Angiogenesis.” Interestingly, and in complete agreement with our previous studies (4, 21), biological processes associated with fatty acid oxidation were significantly enriched in the selected genes. Some important genes related to these biological processes were *BDH2, ECHS1, AUH* and *CYP4V2* (Supplementary material S5). *BDH2*, 3-hydroxybutyrate dehydrogenase 2, is involved in ketogenesis (synthesis and degradation of ketone bodies) and can stimulate fat storage and adiposity by providing precursors for lipid and sterol synthesis (58). On the other hand, role of this gene in fatty acid oxidation has been highlighted in previous studies (59, 60). The second step of the *β*-oxidation pathway is catalyzed by *ECHS1*. In this regard, up-regulation of this gene is reported to be associated with a reduction in abdominal fat deposition in broilers via the promotion of fatty acid oxidation (61). It was demonstrated that the expression of *ECHS1* in the liver tissue of mice fed with high fat diet decreased and led to accelerated acetyl coenzyme production, which is the precursor of fatty acid synthesis (62). These findings provide additional support for our previous hypothesis, which suggests that the genes involved in fatty acid oxidation May promote this biological process and modulate fat accumulation in the tail of sheep breeds. Considering that fat-tailed breeds originate from thin-tailed sheep, it can be hypothesized that harsh environmental conditions May have led to both artificial and natural selection, favoring alleles that downregulate the genes involved in fatty acid oxidation in the tail of sheep. It is worth noting that none of the discussed genes were previously reported as candidate genes involved in fat-tail development in sheep and can be considered novel genes associated with this trait. Regarding the “Sprouting Angiogenesis” process, it is well documented that development of obesity is associated with adipogenesis and angiogenesis (63–65). The process of forming new blood vessels, angiogenesis, is reported as a rate-limiting step in fat tissue expansion. In this regard, vasculature is responsible for the transport of fatty acids to other tissues as well as supplies oxygen and nutrients, which is necessary for fat tissue expansion (64). *VEGF* (Vascular endothelial growth factors) family members are considered to be the main angiogenic components in adipose tissue and showed high expression in adipocytes and adipose tissue of obese mice (66). An important member of this family is *VEGFD* (detected in the candidate regions in the present study), which play a crucial role in development of adipose tissue (65). Furthermore, “Cell Surface Receptor Signaling Pathway Involved In Cell–Cell Signaling “was a significant functional term, which refers to a series of molecular signals initiated when an extracellular ligand binds to a receptor on the cell surface. It then triggers a cascade of events inside the cell and ultimately regulating downstream cellular processes, such as lipid metabolism. In this context, the development of adipose tissue through cell–cell signaling has been documented (67). BMP2 as a gene involved in this pathway, has been suggested to be associated with depot-specific preadipocyte development and abdominal AT expansion in humans via BMP2-SMAD1/5/8 signaling pathway (68). The other important gene in this pathway is DDX3X, which is known as a potential key regulator of adiposity (69).

To confirm the previously suggested selection signatures and identify novel candidates, a comprehensive literature review of similar studies, focusing on selection signature identification analysis between fat- and thin-tailed sheep breeds, was performed. Among the 87 genes in the selected regions (with gene symbols), 33 genes were reported as candidate genes involved in fat-tail development in previous studies (Supplementary material S4). First, the consistency between our results (inferred from RNA-Seq data) and the previous findings (based on DNA-Seq/Array) emphasize that RNA-Seq datasets are beneficial for population genetics, especially selection signature identification analysis. On the other hand, among the previously reported genes, *BMP2* was of great interest, as it has been reported to be associated with fat-tail development in most previous cohort studies. Bone morphogenetic proteins (BMPs) family members are known for their crucial roles in bone morphogenesis. Numerous studies have highlighted the role of *BMP2* in fat deposition as well as the regulation of adipogenesis metabolism (8, 10, 13, 16, 70–74). In perfect agreement with these studies, our results emphasized the potential role of *BMP2* as the most promising candidate associated with fat-tail morphology in sheep. In contrast, *PDGFD* is reported as the predominant factor for the fat tail phenotype in sheep and it is not detected in our results (75). It can be attributed to many reasons including average/low density of the identified SNPs by RNA-Seq data, inadequate sample size or the methods that used to detect the signatures. Further investigation of the identified genes indicated that fat-tail morphology in sheep might be explained by diverse regulatory mechanisms. Hence, it is worthwhile to highlight other important genes that are reported to be directly associated with lipid metabolism, such as *RGMA, IGFBP7, UBR5, WLS* and *NID2. RGMA* mediates the expression of peroxisome proliferator-activated receptor (*PPARγ*), a key modulator of lipid metabolism (76). Additionally, *RGMA* is associated with the BMP signaling pathway. It has been demonstrated that this pathway is relevant for both white and brown adipogenesis (77). The role of *IGFBP7* as a regulator of abnormal lipid metabolism has been reported (78). *UBR5* and *WLS* are involved in Wnt signaling, which is a well-known and main regulator of adipogenesis (79). It has been suggested that *NID2* can regulate adipogenesis and body fat distribution (80). These findings clearly showed that the effect of selection on the genes involved in beta-oxidation of fatty acids is not the only way in which selection shaped the genetic profile of sheep to adapt to various and often food shortage conditions. Hence, the genes described above, especially *BMP2*, can be considered candidates involved in fat-tail development during the evolution of sheep breeds and can also be considered as a basis for subsequent studies of related traits.

PCA analysis showed robust separation between the two sheep groups, revealing a high degree of similarity among the samples within each group (fat- and thin-tailed groups). This clear separation between the two groups May suggest a common genetic origin for each group. In this context, it is well established that the wild ancestor of domestic sheep, the Mouflon sheep, is thin-tailed. Consequently, modern thin-tailed sheep are expected to show the ancestral allele state, whereas fat-tailed sheep exhibit derived alleles at loci associated with the fat tail phenotype (75). Based on the variance explained by each PC, 629 and six important SNPs were found to contribute to PC1 and PC2 and were related to 70 and two genes, respectively. Several of these genes (including *CYP4V2, IKBKG, BDH2*, *BMP2*, *ERBIN* and *ECHS1*) were directly annotated to be involved in fat metabolism-related biological processes, and 29 genes were reported to be involved in fat deposition based on previous studies. For instance, *CYP4V2* encodes an enzyme that produces arachidonic acid and plays an important role in fat deposition. This gene influences abdominal fat storage in cattle and pigs (81, 82). Furthermore, a recent study described the role of *CYP4V2* in the accumulation of lipid droplets in retinal pigment epithelium cells in zebrafish (83). The *IKBKG* gene plays an important role in fat metabolism by regulating blood lipid levels, insulin sensitivity and energy consumption (84). These findings reinforced the results of the selection signature identification analysis and suggested strong positive selection at these loci. Together, these results enabled us to explain the genetic basis of fat-tail size in sheep. Here, we attempted to discuss candidate genes that were reported to be associated with fat metabolism. Although other candidate genes do not have direct evidence of involvement in fat metabolism, they are worthy of further investigation. Finally, as this comes as a limitation to our study, it is clear that RNA-Seq data only captures SNPs within expressed gene regions, inherently excludes regulatory variants, introns, and other non-coding regions. Furthermore, higher sample size, particularly the higher number of samples for each breed pair, May improve the statistical power of the findings as well as the generalizability of the results, which we acknowledge as another limitation of this study. While Rambouillet v2 is the latest version of the sheep reference genome at the time of writing this paper, Rambouillet v1 was the latest version at the time of analysis. However, using the new version of genome cannot change the overall results, as Rambouillet v2 improved continuity and no more sequences are added to the genome. In fact, the number of scaffolds is changed in the new version and the genomic sequences of both versions are same.

## 5 Conclusion

In this study, a comparative genomic study (fat- vs. thin-tailed sheep breeds) was conducted based on the identified SNPs from RNA-Seq datasets to discover the genomic signatures that May be linked to fat-tail development. The consistency in the candidate selection signatures between our results and those of previous studies showed the usefulness of RNA-Seq data for SNP calling as well as selection signature analysis. Functional analysis of the genes in the identified selection signature regions highlighted our previous hypothesis that genes associated with fatty acid oxidation May modulate fat deposition in the tail of sheep. Interestingly and in agreement with the previous reports, angiogenesis process was also enriched, which reinforces its potential regulatory role in tail fat deposition of sheep. Furthermore, our findings revealed that the identified genomic regions containing genes directly related to fat metabolism and also previously reported to be associated with the fat-tail phenotype in sheep, such as *BMP2*, *NID2*, *IKBKG*, *RGMA, IGFBP7, UBR5, VEGFD* and *WLS*. In addition, some well-known fat metabolism-associated genes, different from those reported in previous studies, were identified, including *BDH2, ECHS1, AUH, ERBIN* and *CYP4V2.* The results of the functional enrichment analysis along with the PCA provided additional evidence for the importance of the identified regions in fat-tail development. These findings suggest that the identified selection signatures can be considered as specific evolutionary forces responsible for fat-tailed breed adaptation to various environmental conditions, which has also led to a better understanding of sheep evolution across different species.

## Data Availability

The datasets presented in this study can be found in online repositories. The names of the repository/repositories and accession number(s) can be found in the article/Supplementary material.

## References

[1] Taylor WTT Pruvost M Posth C Rendu W Krajcarz MT Abdykanova A. Evidence for early dispersal of domestic sheep into Central Asia. Nat Hum Behav. (2021) 5:1169–79. doi: 10.1038/s41562-021-01083-y, PMID: 33833423

[2] Meadows JRS Chan EKF Kijas JW. Linkage disequilibrium compared between five populations of domestic sheep. BMC Genet. (2008) 9:1–10. doi: 10.1186/1471-2156-9-61/TABLES/4 18826649 PMC2572059

[3] Kalds P Huang S Chen Y Wang X. Ovine HOXB13: expanding the gene repertoire of sheep tail patterning and implications in genetic improvement. Commun Biol. (2022) 5:1196. doi: 10.1038/s42003-022-04199-7, PMID: 36344811 PMC9640552

[4] Bakhtiarizadeh MR Alamouti AA. RNA-Seq based genetic variant discovery provides new insights into controlling fat deposition in the tail of sheep. Sci Reports. (2020) 10:13525. doi: 10.1038/s41598-020-70527-8, PMID: 32782325 PMC7419499

[5] Oleksyk TK Smith MW O’Brien SJ. Genome-wide scans for footprints of natural selection. Philos Trans R Soc B Biol Sci. (2010) 365:185–205. doi: 10.1098/RSTB.2009.0219, PMID: 20008396 PMC2842710

[6] Stephan W. Selective sweeps. Genetics. (2019) 211:5–13. doi: 10.1534/GENETICS.118.301319, PMID: 30626638 PMC6325696

[7] Horscroft C Ennis S Pengelly RJ Sluckin TJ Collins A. Sequencing era methods for identifying signatures of selection in the genome. Brief Bioinform. (2019) 20:1997–2008. doi: 10.1093/BIB/BBY064, PMID: 30053138

[8] Zhao F Deng T Shi L Wang W Zhang Q Du L. Genomic scan for selection signature reveals fat deposition in Chinese indigenous sheep with extreme tail types. Animals. (2020) 10:773. doi: 10.3390/ani10050000 32365604 PMC7278473

[9] Yuan Z Liu E Liu Z Kijas JW Zhu C Hu S. Selection signature analysis reveals genes associated with tail type in Chinese indigenous sheep. Anim Genet. (2017) 48:55–66. doi: 10.1111/AGE.12477 27807880

[10] Moradi MH Nejati-Javaremi A Moradi-Shahrbabak M Dodds KG Brauning R McEwan JC. Hitchhiking mapping of candidate regions associated with fat deposition in Iranian thin and fat tail sheep breeds suggests new insights into molecular aspects of fat tail selection. Animals. (2022) 12:1423. doi: 10.3390/ani12111423, PMID: 35681887 PMC9179914

[11] Moradi MH Nejati-Javaremi A Moradi-Shahrbabak M Dodds KG McEwan JC. Genomic scan of selective sweeps in thin and fat tail sheep breeds for identifying of candidate regions associated with fat deposition. BMC Genet. (2012) 13:10. doi: 10.1186/1471-2156-13-10, PMID: 22364287 PMC3351017

[12] Manzari Z Mehrabani-Yeganeh H Nejati-Javaremi A Moradi MH Gholizadeh M. Detecting selection signatures in three Iranian sheep breeds. Anim Genet. (2019) 50:298–302. doi: 10.1111/AGE.12772, PMID: 30883840

[13] Mastrangelo S Moioli B Ahbara A Latairish S Portolano B Pilla F. Genome-wide scan of fat-tail sheep identifies signals of selection for fat deposition and adaptation. Anim Prod Sci. (2018) 59:835–48. doi: 10.1071/AN17753

[14] Al-Mamun HA Kwan P Clark SA Ferdosi MH Tellam R Gondro C. Genome-wide association study of body weight in Australian merino sheep reveals an orthologous region on OAR6 to human and bovine genomic regions affecting height and weight. Genet Sel Evol. (2015) 47:66. doi: 10.1186/s12711-015-0142-4, PMID: 26272623 PMC4536601

[15] Shao J Pan X Yang Z Nanaei HA Chen L Li R. Allele-specific expression reveals the phenotypic differences between thin- and fat-tailed sheep. J Genet Genomics. (2020) 49:583–6. doi: 10.21203/RS.3.RS-56388/V1 34998977

[16] Lv X Chen W Wang S Cao X Yuan Z Getachew T. Whole-genome resequencing of Dorper and Hu sheep to reveal selection signatures associated with important traits. Anim Biotechnol. (2023) 34:3016–26. doi: 10.1080/10495398.2022.2127409 36200839

[17] Ahbara A Bahbahani H Almathen F Al AM Agoub MO Abeba A. Genome-wide variation, candidate regions and genes associated with fat deposition and tail morphology in Ethiopian indigenous sheep. Front Genet. (2019) 10:387968. doi: 10.3389/FGENE.2018.00699/BIBTEX PMC6334744 30687385

[18] Satam H Joshi K Mangrolia U Waghoo S Zaidi G Rawool S. Next-generation sequencing technology: current trends and advancements. Biol. (2023) 12:997. doi: 10.3390/BIOLOGY12070997 PMC10376292 37508427

[19] Xu SS Gao L Shen M Lyu F. Whole-genome selective scans detect Genes associated with important phenotypic traits in sheep (Ovis aries). Front Genet. (2021) 12:738879. doi: 10.3389/fgene.2021.738879, PMID: 34868210 PMC8637624

[20] Piskol R Ramaswami G Li JB. Reliable identification of genomic variants from RNA-Seq data. Am J Hum Genet. (2013) 93:641–51. doi: 10.1016/J.AJHG.2013.08.008, PMID: 24075185 PMC3791257

[21] Bakhtiarizadeh MR Salehi A Alamouti AA Abdollahi-Arpanahi R Salami A. Deep transcriptome analysis using RNA-seq suggests novel insights into molecular aspects of fat-tail metabolism in sheep. Sci Rep. 9:9203. doi: 10.1038/s41598-019-45665-3 PMC6591244 31235755

[22] Guangli Y Huan Z Shuhong Z Zhiqiang L Fengyi G Guan W., Identification of the genetic basis for the large-tailed phenotypic trait in Han sheep through integrated mRNA and miRNA analysis of tail fat tissue samples. (2020). Available at: https://www.researchsquare.com/article/rs-107294/v1

[23] Ma L Zhang M Jin Y Erdenee S Hu L Chen H. Comparative transcriptome profiling of mRNA and lncRNA related to tail adipose tissues of sheep. Front Genet. (2018) 9:355770. doi: 10.3389/FGENE.2018.00365/BIBTEX PMC6139350 30250481

[24] Yuan Z Ge L Sun J Zhang W Wang S Cao X. Integrative analysis of Iso-Seq and RNA-seq data reveals transcriptome complexity and differentially expressed transcripts in sheep tail fat. PeerJ. (2021) 9:e12454. doi: 10.7717/peerj.12454, PMID: 34760406 PMC8571958

[25] Fan H Hou Y Sahana G Gao H Zhu C Du L. A transcriptomic study of the tail fat deposition in two types of Hulun Buir sheep according to tail size and sex. Animal. (2019) 9:655. doi: 10.3390/ANI9090655 PMC6770480 31491862

[26] Farhadi S Ghias JS Hasanpur K Mohammadi SA Ebrahimie E. Molecular mechanisms of fat deposition: IL-6 is a hub gene in fat lipolysis, comparing thin-tailed with fat-tailed sheep breeds. Arch Anim Breed. (2021) 64:53–68. doi: 10.5194/AAB-64-53-2021, PMID: 34084904 PMC8130542

[27] Jin M Fei X Li T Lu Z Chu M Di R. Oar-miR-432 regulates fat differentiation and promotes the expression of BMP2 in ovine Preadipocytes. Front Genet. (2022) 13:844747. doi: 10.3389/fgene.2022.844747, PMID: 35559046 PMC9087340

[28] Brown J Pirrung M Mccue LA. FQC dashboard: integrates FastQC results into a web-based, interactive, and extensible FASTQ quality control tool. Bioinformatics. (2017) 33:3137–9. doi: 10.1093/BIOINFORMATICS/BTX373, PMID: 28605449 PMC5870778

[29] Bolger AM Lohse M Usadel B. Trimmomatic: a flexible trimmer for Illumina sequence data. Bioinformatics. (2014) 30:2114–20. doi: 10.1093/BIOINFORMATICS/BTU170, PMID: 24695404 PMC4103590

[30] Veeneman BA Shukla S Dhanasekaran SM Chinnaiyan AM Nesvizhskii AI. Two-pass alignment improves novel splice junction quantification. Bioinformatics. (2016) 32:43–9. doi: 10.1093/BIOINFORMATICS/BTV642, PMID: 26519505 PMC5006238

[31] Hosseini SF Bakhtiarizadeh MR Salehi A. Meta-analysis of RNA-Seq datasets highlights novel genes/pathways involved in fat deposition in fat-tail of sheep. Front Vet Sci. (2023) 10:1159921. doi: 10.3389/fvets.2023.1159921, PMID: 37252399 PMC10213422

[32] Bathke J Lühken G. OVarFlow: a resource optimized GATK 4 based open source variant calling workFlow. BMC Bioinform. (2021) 22:1–18. doi: 10.1186/S12859-021-04317-Y/FIGURES/4 PMC8361789 34388963

[33] Ebbert MTW Wadsworth ME Staley LA Hoyt KL Pickett B Miller J. Evaluating the necessity of PCR duplicate removal from next-generation sequencing data and a comparison of approaches. BMC Bioinform. (2016) 17:491–500. doi: 10.1186/S12859-016-1097-3/FIGURES/6 PMC4965708 27454357

[34] Deng J Xie XL Wang DF Zhao C Lv FH Li X. Paternal origins and migratory episodes of domestic sheep. Curr Biol. (2020) 30:4085–4095.e6. doi: 10.1016/J.CUB.2020.07.077, PMID: 32822607

[35] Yang W Yang Y Zhao C Yang K Wang D Yang J. Animal-ImputeDB: a comprehensive database with multiple animal reference panels for genotype imputation. Nucleic Acids Res. (2020) 48:D659–67. doi: 10.1093/NAR/GKZ854, PMID: 31584087 PMC6943029

[36] LiftOver. Genome Analysis Wiki (2023). Available at: https://genome.sph.umich.edu/wiki/LiftOver (accessed December 19, 2023)

[37] Browning BL Tian X Zhou Y Browning SR. Fast two-stage phasing of large-scale sequence data. Am J Hum Genet. (2021) 108:1880–90. doi: 10.1016/J.AJHG.2021.08.005 34478634 PMC8551421

[38] Browning BL Zhou Y Browning SR. A one-penny imputed genome from next-generation reference panels. Am J Hum Genet. (2018) 103:338–48. doi: 10.1016/J.AJHG.2018.07.015, PMID: 30100085 PMC6128308

[39] Yun L Willer C Sanna S Abecasis G. Genotype imputation. Annu Rev Genomics Hum Genet. (2009) 10:387–406. doi: 10.1146/ANNUREV.GENOM.9.081307.164242 19715440 PMC2925172

[40] Marchini J Howie B. Genotype imputation for genome-wide association studies. Nat Rev Genet. (2010) 11:499–511. doi: 10.1038/nrg2796 20517342

[41] Yin Y Hou L Liu C Li K Guo H Niu P. Genome-wide association study identified a quantitative trait locus and two candidate Genes on Sus scrofa chromosome 2 affecting vulvar traits of Suhuai pigs. Genes (Basel). (2022) 13:1294. doi: 10.3390/genes13081294, PMID: 35893031 PMC9330916

[42] Hao K Chudin E McElwee J Schadt EE. Accuracy of genome-wide imputation of untyped markers and impacts on statistical power for association studies. BMC Genet. (2009) 10:1–10. doi: 10.1186/1471-2156-10-27/FIGURES/4 19531258 PMC2709633

[43] MacEachern S Hayes B McEwan J Goddard M. An examination of positive selection and changing effective population size in Angus and Holstein cattle populations (Bos taurus) using a high density SNP genotyping platform and the contribution of ancient polymorphism to genomic diversity in domestic cattle. BMC Genomics. (2009) 10:1–19. doi: 10.1186/1471-2164-10-181/TABLES/6 19393053 PMC2681480

[44] Weir BS Cockerham CC. Estimating F-statistics for the analysis of population structure. Evolution (N Y). (1984) 38:1358. doi: 10.2307/2408641 28563791

[45] Gholami M Erbe M Gärke C Preisinger R Weigend A Weigend S. Population genomic analyses based on 1 million SNPs in commercial egg layers. PLoS One. (2014) 9:e94509. doi: 10.1371/JOURNAL.PONE.0094509, PMID: 24739889 PMC3989219

[46] Cingolani P Platts A Wang LL Coon M Nguyen T Wang L. A program for annotating and predicting the effects of single nucleotide polymorphisms, SnpEff. Fly (Austin). (2012) 6:80–92. doi: 10.4161/FLY.19695, PMID: 22728672 PMC3679285

[47] Chen EY Tan CM Kou Y Duan Q Wang Z Meirelles GV. Enrichr: interactive and collaborative HTML5 gene list enrichment analysis tool. BMC Bioinform. (2013) 14:128. doi: 10.1186/1471-2105-14-128, PMID: 23586463 PMC3637064

[48] Merkin J Russell C Chen P Burge CB. Evolutionary dynamics of gene and isoform regulation in mammalian tissues. Science. (2012) 338:1593–9. doi: 10.1126/SCIENCE.1228186/SUPPL_FILE/MERKIN.SM.PDF 23258891 PMC3568499

[49] Chan ET Quon GT Chua G Babak T Trochesset M Zirngibl RA. Conservation of core gene expression in vertebrate tissues. J Biol. (2009) 8:1–17. doi: 10.1186/JBIOL130/FIGURES/8 PMC2689434 19371447

[50] Gutiérrez-Gil B Arranz JJ Wiener P. An interpretive review of selective sweep studies in Bos taurus cattle populations: identification of unique and shared selection signals across breeds. Front Genet. (2015) 6:167. doi: 10.3389/FGENE.2015.00167/FULL 26029239 PMC4429627

[51] Muigai AWT Hanotte O. The origin of African sheep: archaeological and genetic perspectives. African Archaeol Rev. (2013) 30:39–50. doi: 10.1007/s10437-013-9129-0

[52] Cadzow M Boocock J Nguyen HT Wilcox P Merriman TR Black MA. A bioinformatics workflow for detecting signatures of selection in genomic data. Front Genet. (2014) 5:102714. doi: 10.3389/FGENE.2014.00293/ABSTRACT PMC4144660 25206364

[53] Yang J Li WR Lv FH He SG Tian SL Peng WF. Whole-genome sequencing of native sheep provides insights into rapid adaptations to extreme environments. Mol Biol Evol. (2016) 33:2576–92. doi: 10.1093/MOLBEV/MSW129, PMID: 27401233 PMC5026255

[54] Ai H Fang X Yang B Huang Z Chen H Mao L. Adaptation and possible ancient interspecies introgression in pigs identified by whole-genome sequencing. Nat Genet. (2015) 47:217–25. doi: 10.1038/ng.3199, PMID: 25621459

[55] Zhang S Yao Z Li X Zhang Z Liu X Yang P. Assessing genomic diversity and signatures of selection in Pinan cattle using whole-genome sequencing data. BMC Genomics. (2022) 23:1–10. doi: 10.1186/S12864-022-08645-Y/TABLES/1 35729510 PMC9215082

[56] Shafiei H Bakhtiarizadeh MR Salehi A. Large-scale potential RNA editing profiling in different adult chicken tissues. Anim Genet. (2019) 50:460–74. doi: 10.1111/AGE.12818, PMID: 31355950

[57] Bakhtiarizadeh MR Salehi A Rivera RM. Genome-wide identification and analysis of A-to-I RNA editing events in bovine by transcriptome sequencing. PLoS One. (2018) 13:e0193316. doi: 10.1371/JOURNAL.PONE.0193316, PMID: 29470549 PMC5823453

[58] Bonnet A Cao KL Sancristobal M. In vivo gene expression in granulosa cells during pig terminal follicular development. Reproduction. (2008) 136:211–24. doi: 10.1530/REP-07-0312 18456903

[59] Kamei A Watanabe Y Kondo K Okada S Shinozaki F Ishijima T. Influence of a short-term Iron-deficient diet on hepatic gene expression profiles in rats. PLoS One. (2013) 8:e65732. doi: 10.1371/JOURNAL.PONE.0065732, PMID: 23755274 PMC3674005

[60] Wang L Zhang Y Zhang B Zhong H Lu Y Zhang H. Candidate gene screening for lipid deposition using combined transcriptomic and proteomic data from Nanyang black pigs. BMC Genomics. (2021) 22:441. doi: 10.1186/S12864-021-07764-2, PMID: 34118873 PMC8201413

[61] Peng M Han J Li L Reports HM-S. Suppression of fat deposition in broiler chickens by (−)-hydroxycitric acid supplementation: A proteomics perspective (2016). Available at: https://www.nature.com/articles/srep32580 (accessed December 17, 2023). 10.1038/srep32580 PMC5009311 27586962

[62] Fan C Hu H Huang X Su D Huang F Zhuo Z. Betaine supplementation causes an increase in fatty acid oxidation and carbohydrate metabolism in livers of mice fed a high-fat diet: A proteomic analysis. Food Secur. (2022) 11:881. doi: 10.3390/FOODS11060881 PMC8949908 35327303

[63] Alonso-García M Suárez-Vega A Fonseca PAS Marina H Pelayo R Mateo J. Transcriptome analysis of perirenal fat from Spanish Assaf suckling lamb carcasses showing different levels of kidney knob and channel fat. Front Vet Sci. (2023) 10:1150996. doi: 10.3389/fvets.2023.1150996, PMID: 37255997 PMC10225515

[64] Alonso-García M Gutiérrez-Gil B Pelayo R Fonseca PAS Marina H Arranz JJ. A meta-analysis approach for annotation and identification of lncRNAs controlling perirenal fat deposition in suckling lambs. Anim Biotechnol. (2024) 35:2374328. doi: 10.1080/10495398.2024.2374328, PMID: 39003576 PMC12674383

[65] Lijnen H Frederix L Van Hoef B Dewerchin M. Deficiency of vascular endothelial growth factor-D does not affect murine adipose tissue development (2009). Available at: https://www.sciencedirect.com/science/article/pii/S0006291X08022225 (accessed September 2, 2024). 10.1016/j.bbrc.2008.11.032 19022221

[66] Voros G Maquoi E Demeulemeester D Clerx N Collen D Lijnen HR. Modulation of angiogenesis during adipose tissue development in murine models of obesity. Endocrinology. (2005) 146:4545–54. doi: 10.1210/EN.2005-0532, PMID: 16020476

[67] Khan T Muise ES Iyengar P Wang ZV Chandalia M Abate N. Metabolic dysregulation and adipose tissue fibrosis: role of collagen VI. Mol Cell Biol. (2009) 29:1575–91. doi: 10.1128/mcb.01300-08, PMID: 19114551 PMC2648231

[68] Denton NF Eghleilib M Al-Sharifi S Todorčević M Neville MJ Loh N. Bone morphogenetic protein 2 is a depot-specific regulator of human adipogenesis. Int J Obes. (2019) 43:2458–68. doi: 10.1038/s41366-019-0421-1, PMID: 31324879 PMC6892741

[69] Savva C Helguero LA González-Granillo M Melo T Couto D Buyandelger B. Maternal high-fat diet programs white and brown adipose tissue lipidome and transcriptome in offspring in a sex- and tissue-dependent manner in mice. Int J Obes. (2022) 46:831–42. doi: 10.1038/s41366-021-01060-5, PMID: 34997206 PMC8960419

[70] Moioli B Pilla F Ciani E. Signatures of selection identify loci associated with fat tail in sheep. J Anim Sci. (2015) 93:4660–9. doi: 10.2527/jas.2015-9389 26523558

[71] Deniskova T Kunz E Medugorac I. A study of genetic mechanisms underlying the fat tail phenotype in sheep: methodological approaches and identified candidate genes (2019) Available at: http://www.agrobiology.ru/articles/6-2019deniskova.pdf (accessed December 17, 2023).

[72] Zhang C Zhang J Tuersuntuoheti M Zhou W Han Z Li X. Landscape genomics reveals adaptive divergence of indigenous sheep in different ecological environments of Xinjiang, China. Sci Total Environ. (2023) 904:166698. doi: 10.1016/j.scitotenv.2023.166698 37683864

[73] Bedhiaf-Romdhani S Baazaoui I Dodds KG Brauning R Anderson RM Van Stijn TC. Efficiency of genotyping by sequencing in inferring genomic relatedness and molecular insights into fat tail selection in Tunisian sheep. Anim Genet. (2023) 54:389–97. doi: 10.1111/AGE.13296, PMID: 36727208

[74] Baazaoui I Bedhiaf-Romdhani S Animal SM. Genome-wide analyses reveal population structure and identify candidate genes associated with tail fatness in local sheep from a semi-arid area. Animal. (2021) 15:100193. doi: 10.1016/j.animal.2021.100193 33715983

[75] Dong K Yang M Han J Ma Q Han J Song Z. Genomic analysis of worldwide sheep breeds reveals PDGFD as a major target of fat-tail selection in sheep. BMC Genomics. (2020) 21:1–12. doi: 10.1186/S12864-020-07210-9/FIGURES/4 PMC7670677 33203382

[76] Yuan H Suzuki S Hirata-Tsuchiya S Sato A Nemoto E Saito M. PPARγ-induced global H3K27 acetylation maintains osteo/cementogenic abilities of periodontal ligament fibroblasts. Int J Mol Sci. (2021) 22:8646. doi: 10.3390/ijms22168646 34445348 PMC8395443

[77] Mir FA Mall R Iskandarani A Ullah E Samra TA Cyprian F. Characteristic MicroRNAs linked to dysregulated metabolic pathways in Qatari adult subjects with obesity and metabolic syndrome. Front Endocrinol (Lausanne). (2022) 13:937089. doi: 10.3389/fendo.2022.937089, PMID: 35937842 PMC9352892

[78] Stanley T Fourman L McClure CM Feldpausch MN Torriani M Corey KE. Relationship of IGF-1 and IGF-binding proteins to disease severity and glycemia in nonalcoholic fatty liver disease. J Clin Endocrinol Metab. (2021) 106:e520–33. doi: 10.1210/clinem/dgaa792 33125080 PMC7823253

[79] Song K Wang S Mani M Mani A. Wnt signaling, de novo lipogenesis, adipogenesis and ectopic fat. Oncotarget. (2014) 5:11000–3. doi: 10.18632/oncotarget.2769 25526027 PMC4294374

[80] Sun C Kovacs P Genes EG-J. Genetics of body fat distribution: comparative analyses in populations with European, Asian and African ancestries. Genes (Basel). (2021, 2021) 12:841. doi: 10.3390/genes12060841 34072523 PMC8228180

[81] Zhang Y Sun Y Wu Z Xiong X Zhang J Ma J. Subcutaneous and intramuscular fat transcriptomes show large differences in network organization and associations with adipose traits in pigs. Sci China Life Sci. (2021) 64:1732–46. doi: 10.1007/s11427-020-1824-7, PMID: 33527326

[82] Schumacher ML Boles JA Thomson JM. A comparative approach to refine molecular mechanisms impacting meat quality and carcass characteristics. Transl Anim Sci. (2021) 5:S189–94. doi: 10.1093/TAS/TXAB184

[83] Gao P Jia D Li P Huang Y Hu H Sun K. Accumulation of lipid droplets in a novel Bietti crystalline dystrophy zebrafish model with impaired PPARα pathway. Invest Ophthalmol Vis Sci. (2022) 63:32–2. doi: 10.1167/IOVS.63.5.32, PMID: 35616930 PMC9150832

[84] Breda S Van WL DJ GS. The exposome concept in a human nutrigenomics study: evaluating the impact of exposure to a complex mixture of phytochemicals using transcriptomics signatures. Mutagenesis. (2015) 30:723–31. doi: 10.1093/mutage/gev008 25711498

