# Supplementary material for: RNA-Seq based selection signature analysis for identifying genomic footprints associated with the fat-tail phenotype in sheep
Source: Front Vet Sci. 2024 Sep 30;11:1415027. doi: 10.3389/fvets.2024.1415027 (PMC11471730; doi:10.3389/fvets.2024.1415027)

Supplementary Image S1. Correlation of F_{ST} coefficients by Wright's method and unbiased theta coefficients by Weir and Cockerham's method

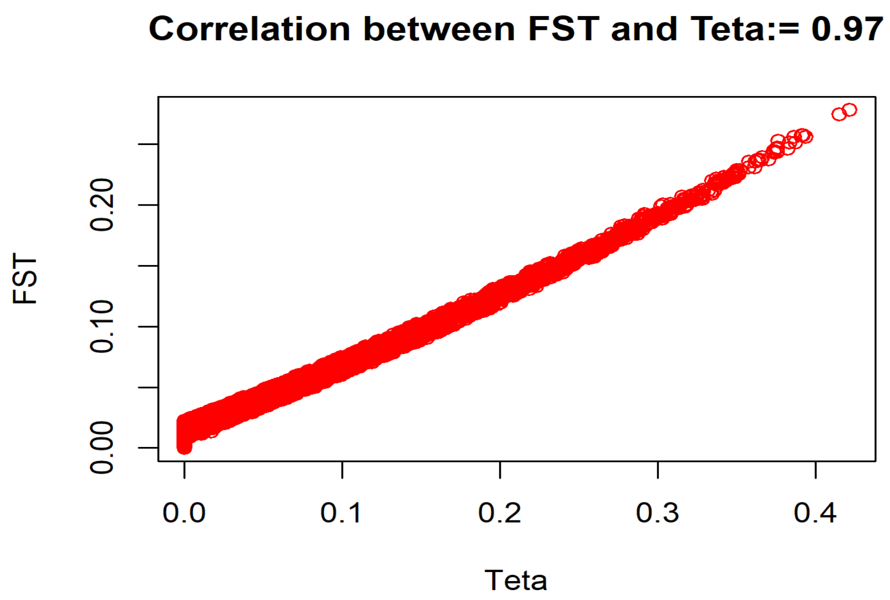

Supplement: Supplementary file 1 [file Image_1.pdf]
